# A retrospective study of ^68^Ga-FAPI PET/CT in differentiating the nature of pulmonary lesions

**DOI:** 10.3389/fonc.2024.1373286

**Published:** 2024-05-08

**Authors:** Yang Xie, Wenxin Tang, Jiao Ma, Yue Chen

**Affiliations:** ^1^ Department of Nuclear Medicine, The Affiliated Hospital of Southwest Medical University, Luzhou, Sichuan, China; ^2^ Nuclear Medicine and Molecular Imaging Key Laboratory of Sichuan Province, Luzhou, Sichuan, China; ^3^ Academician (Expert) Workstation of Sichuan Province, Luzhou, Sichuan, China; ^4^ Department of Neurology, The Affiliated Hospital of Southwest Medical University, Luzhou, Sichuan, China; ^5^ Department of Nuclear Medicine, Zhongshan Hospital, Fudan University, Shanghai, China; ^6^ Institute of Nuclear Medicine, Fudan University, Shanghai, China

**Keywords:** pulmonary lesions, 68 Ga-fibroblast activating protein inhibitor (FAPI) -04, positron emission tomography, Gallium radioisotopes, molecular imaging

## Abstract

**Purpose:**

This study aimed to investigate the characteristics of various pulmonary lesions as revealed by ^68^Ga-FAPI PET/CT and to determine the utility of ^68^Ga-FAPI PET/CT in distinguishing the nature of these pulmonary lesions.

**Methods:**

A retrospective analysis was conducted on 99 patients with pulmonary lesions, who were categorized into three distinct groups: primary lung tumors (G1), metastatic lung tumors (G2), and benign lesions (G3). Each participant underwent a ^68^Ga-FAPI PET/CT scan. Among these groups, variables such as the Tumor/Background Ratio (TBR), Maximum Standardized Uptake Value (SUVmax), and the true positive rate of the lesions were compared. Furthermore, the FAPI uptake in nodular-like pulmonary lesions (d<3cm) and those with irregular borders was evaluated across the groups. A correlation analysis sought to understand the relationship between FAPI uptake in primary and pulmonary metastatic lesions.

**Results:**

The study’s participants were composed of 52 males and 47 females, with an average age of 56.8 ± 13.2 years. A higher uptake and detection rate for pulmonary lesions were exhibited by Group G1 compared to the other groups (SUVmax [G1 vs. G2 vs. G3: 9.1 ± 4.1 vs. 6.1 ± 4.1 vs. 5.3 ± 5.8], *P*<0.05; TBR [G1 vs. G2 vs. G3: 6.2 ± 2.4 vs. 4.1 ± 2.2 vs. 3.2 ± 2.7], *P*<0.01; true positive rate 95.1% vs. 88% vs. 75.6%]. In nodular-like lung lesions smaller than 3 cm, G1 showed a significantly higher FAPI uptake compared to G2 and G3 (SUVmax [G1 vs. G2 vs. G3: 8.8 ± 4.3 vs. 5.2 ± 3.2 vs. 4.9 ± 6.1], *P*<0.01; TBR [G1 vs. G2 vs. G3: 5.7 ± 2.7 vs. 3.7 ± 2.1 vs. 3.3 ± 4.4], *P*<0.05). Both G1 and G2 demonstrated significantly elevated FAPI agent activity in irregular-bordered pulmonary lesions when compared to G3 (SUVmax [G1 vs. G2 vs. G3: 10.9 ± 3.3 vs. 8.5 ± 2.7 vs. 4.6 ± 2.7], *P*<0.01; TBR [G1 vs. G2 vs. G3: 7.2 ± 2.1 vs. 6.4 ± 1.3 vs. 3.2 ± 2.4], *P*<0.01). A positive correlation was identified between the level of ^68^Ga-FAPI uptake in primary lesions and the uptake in pulmonary metastatic lesions within G2 (r=0.856, *P*<0.05).

**Conclusion:**

^68^Ga-FAPI PET/CT imaging proves to be of significant value in the evaluation of pulmonary lesions, offering distinctive insights into their nature.

## 1 Introduction

Lung cancer remains the leading cause of cancer-related mortality globally (1). Beyond primary lung cancer, malignant tumor lung metastases significantly contribute to lung malignancy incidences. Malignant tumors originating from organs like the gastrointestinal tract, breast, and liver frequently metastasize to the lungs. For patients with known primary tumors in other organs accompanied by lung lesions, accurately characterizing these lung lesions is crucial for determining the staging of the primary tumor and guiding subsequent clinical treatment decisions (2). Historically, traditional imaging techniques have struggled to distinguish the nature of lung lesions based solely on their morphological characteristics. The widespread adoption of chest CT for lung cancer screening, accelerated by the novel corona-virus pneumonia pandemic, has highlighted this challenge. Morphological features observed in benign pulmonary conditions such as inflammation, organizing pneumonia, and tuberculous granulomas on chest CT can often mimic those of malignant lung tumors (3, 4), thereby limiting the utility of chest CT, which predominantly relies on morphological criteria for diagnosing pulmonary lesions (5). Although invasive procedures like puncture biopsy, surgical resection, and pathological examination are definitive methods for diagnosing pulmonary lesions, they pose risks of complications, including pneumothorax, hemoptysis, and intracranial air embolism (6). Consequently, the exploration of non-invasive imaging techniques for analyzing the nature of pulmonary lesions holds significant importance in minimizing the need for unnecessary invasive treatments.

FAPI, which binds specifically to Fibroblast Activation Protein (FAP) on the cell membrane, can be labeled with radionuclides for use in PET/CT imaging (7–9). Given the high expression of FAP in more than 90% of epithelial histogenic tumors, radionuclide-labeled FAPI has demonstrated significant potential in the diagnosis of such tumors (10). Gallium ^68^ (^68^Ga)-labeled FAPI has shown high affinity and favorable target-to-nontarget (T/NT) ratios in PET/CT imaging across a variety of tumors (11, 12). With the increasing clinical adoption of ^68^Ga-FAPI PET/CT, reports have highlighted its elevated uptake in various benign conditions, including benign nodular diseases of the lungs such as organizing pneumonia, pulmonary tuberculosis, and Klebsiella pneumonia invasive syndrome (13–15). Research into the diagnostic efficacy of ^68^Ga-FAPI PET/CT imaging for lung lesions has predominantly concentrated on distinguishing specific types of primary pulmonary tumors or metastases from other solid organs to the lungs, like lung cancer and breast cancer metastases to the lungs. Since both ^68^Ga-FAPI and 18F-FDG act as broad-spectrum tracers, assessing their capacity to differentiate between benign and malignant lung lesions could offer valuable insights for tracer selection in PET/CT imaging, thus aiding in the determination of the nature of lung lesions in clinical patients. Therefore, the objective of this study was to preliminary evaluate the efficacy of ^68^Ga-FAPI PET/CT imaging in distinguishing the nature of pulmonary lesions through a retrospective analysis and to investigate its potential in differentiating primary malignant lesions, metastases, and benign pulmonary lesions.

## 2 Methods

### 2.1 Patients

This study included 99 patients who presented with pulmonary lesions and underwent ^68^Ga-FAPI PET/CT scans between January 2021 and August 2023. These patients were classified into three groups: the primary tumor group (G1, consisting of 41 patients), the metastasis group (G2, comprising 25 patients), and the benign lesion group (G3, with 33 patients). All participants met the specified inclusion and exclusion criteria. The inclusion criteria were as follows (1): Positive pulmonary lesions detected through ^68^Ga-FAPI PET/CT imaging (2). The lesion’s nature is confirmed via pathological methods (e.g. tissue sectioning, immunohistochemical staining), potentially supplemented by clinical interventional imaging follow-up and laboratory examination results supporting the diagnosis (3). The acquisition of patient informed consent. The exclusion criteria included (1): Absence of positive pulmonary lesions in ^68^Ga-FAPI PET/CT imaging or the presence of positive lesions whose SUVmax could not be measured (2). Inadequate clinical, pathological, imaging, and laboratory data to accurately determine the nature of the pulmonary lesion (3). Patient refusal to participate in the study. All participants who met the inclusion criteria signed an informed consent form for the ^68^Ga-FAPI PET/CT procedure. This retrospective study received approval from the Ethics Committee of the Affiliated Hospital of Southwest Medical University.

### 2.2 Drug preparation

The radioactive isotope ^68^Ga, used for labeling, was obtained by elution from the Germanium-Gallium generator (ITG, Germany). The FAPI-04 precursor, acquired from MCE (MedChem Express, USA), exhibits a purity exceeding 98%. These components are combined and heated at 95°C for 15 minutes to produce the target compound, ^68^Ga-FAPI-04. This entire procedure is conducted under aseptic conditions, with quality control assured through high-performance liquid chromatography (HPLC, Lab Alliance), achieving a radiochemical purity greater than 99%.

### 2.3 ^68^Ga-FAPI PET/CT imaging

An intravenous injection of ^68^Ga-FAPI tracer at a dose of 1.85 MBq/kg was administered to patients, and imaging was conducted 45-60 minutes after injection using the United Imaging UMI780 PET/CT scanner. The CT scan spanned from the skull base to mid-thigh, employing parameters of 120 kV tube voltage, 120 mA tube current, and a slice thickness of 3.00 mm. The subsequent PET scan was executed in the identical bed position as the CT, utilizing a three-dimensional acquisition mode across 5-6 bed positions with a duration of 3 minutes per position. All PET images underwent iterative reconstruction (Ordered-subset Expectation Maximization (OSEM)) and were analyzed using United Imaging’s proprietary post-processing fusion software.

### 2.4 Image analysis

The ^68^Ga-FAPI-04 images were analyzed by two experienced nuclear medicine physicians who reached a consensus on the findings. A positive uptake of ^68^Ga-FAPI-04 was identified as a focal uptake exceeding background levels or visually apparent in adjacent areas, in line with the known distribution patterns of imaging tracers *in vivo*. For Standardized Uptake Value (SUV) calculations, the SUVmax was documented in the transaxial position utilizing the United Imaging after processing workstation (uWS-MI, Version R002, United Imaging, Shanghai, China). A technician, boasting 8 years of experience, delineated the 3D Region of Interest (ROI) for the lesions on PET/CT images automatically, based on 40% of the maximum threshold value. Additionally, a sphere with a 1 cm diameter surrounding the lesions, identified as non-lesional areas, served as the background for manual delineation. The SUVmax for all lesions was divided by the corresponding background SUVmean to determine the TBR. For CT image analysis, both the size and shape of lung lesions were assessed in accordance with the American College of Chest Physicians (ACCP) and the Fleischner Society guidelines, which denote lesion size and shape as independent risk factors. In cases presenting multiple lung lesions, two criteria were applied: (1) a higher SUVmax value; and (2) lesion nature confirmed by pathological biopsy or follow-up. When criterion 2 was satisfied, the lesion meeting criterion 1 was selected for analysis.

### 2.5 Statistical analysis

All statistical analyses were executed utilizing SPSS 26.0 software (IBM, NY, USA). Categorical variables are presented as frequencies and percentages, while continuous variables are expressed as means ± Standard Deviation (SD). The normality of continuous variables was evaluated using the Shapiro-Wilk test. Differences in TBR values and SUVmax among the three groups were analyzed using the One-Way ANOVA test. For binary data, such as differences in lesion size and shape among groups, the χ2 test and One-Way ANOVA were applied. The Fisher exact probability method was utilized to assess the differences in the true positive rate of tracer uptake in lung lesions across groups. Pearson correlation analysis was conducted to examine the relationship between radiotracer uptake levels in primary lesions and pulmonary metastatic lesions. *P*<0.05 (two-tailed) was deemed to indicate statistical significance.

## 3 Results

### 3.1 Patients characteristics

The study enrolled 99 patients, with an average age of 56.8 ± 13.2 years (range 16-83 years), comprising 42 males and 37 females (**Table 1**). Participants were categorized into three groups based on the nature of their pulmonary lesions: the primary lung tumor group (G1, n=41, average age: 58.4 ± 12.8 years), the metastatic lung tumor group (G2, n=25, average age: 53.0 ± 13.0 years), and the benign lesion group (G3, n=33, average age: 57.8 ± 13.7 years). Age and gender distribution among the groups showed no significant differences (F=1.48; *P*>0.05). All participants underwent ^68^Ga-FAPI PET/CT scans. The disease types within each group are detailed in **Table 2**. Group G1 comprised non-small cell lung cancer (n=35, 85.4%), small cell lung cancer (n=4, 9.7%), and solitary fibrous tumor (n=2, 4.9%). Group G2 included primary tumor sources from the thyroid (n=9, 36.0%), gastrointestinal tract (n=6, 24.0%), pancreas (n=3, 12.0%), lymphoma lung infiltration (n=2, 8.0%), breast (n=1, 4.0%), pleura (n=1, 4.0%), cervix (n=1, 4.0%), liver (n=1, 4.0%), and nasopharynx (n=1, 4.0%). Group G3 consisted of bacterial pneumonia (n=16, 48.5%), tuberculosis (n=4, 12.3%), granuloma (n=3, 9.0%), fungal pneumonia (n=3, 9.0%), pneumoconiosis (n=3, 9.0%), fibrous tissue proliferation (n=2, 6.1%), and organizing pneumonia (n=2, 6.1%).

**Table 1:** Summary of patient characteristics.

Characteristics	Value			
	Total	G1	G2	G3
No. of participants	99	41	25	33
Gender				
Male	42	21	10	21
Female	37	20	15	12
Age (y)				
Mean ± standard deviation	56.8±13.2	58.4±12.8	53.0±13.0	57.8±13.7
Interquartile range	16-83	24-78	16-76	25-83
Size				
Mass-like lesion(d>3cm)	19/99(19.2%)	9/41(21.9%)	5/25(20.0%)	5/33 (15.2%)
Nodular pulmonary lesions (d<3cm)	80/99(80.8%)	32/41(78.1%)	20/25(80.0%)	28/33 (84.8% )
Morphology				
Irregular-bordered (No.)	54/99 (54.5%)	31/41 (75.6%)	5/25(20.0%)	18/33(54.5%)
Lesion with smooth margins (No.)	45/99 (45.5%)	10/41 (24.4%)	20/25 (80.0%)	15/33 (45.5%)

**Table 2:** Summarize the types of diseases in each group.

Types of diseases	Group	No.	%
Primary pulmonary tumor	G1	41	
Non-small cell lung cancer	G1	35	85.4
Small cell lung cancer	G1	4	9.7
Solitary fibrous tumor	G1	2	4.9
Metastatic pulmonary tumor (Primary tumor source)	G2	25	
Thyroid gland	G2	9	36.0
Gastrointestinal tract	G2	6	24.0
Pancreas	G2	3	12.0
Lymphoma lung infiltration	G2	2	8.0
Breast	G2	1	4.0
Pleura	G2	1	4.0
Cervix	G2	1	4.0
Liver	G2	1	4.0
Nasopharynx	G2	1	4.0
Benign pulmonary disease	G3	33	
Bacterial pneumonia	G3	16	48.5
Tuberculosis	G3	4	12.3
Granuloma	G3	3	9.0
Fungal pneumonia	G3	3	9.0
Pneumoconiosis	G3	3	9.0
Fibrous tissue proliferation	G3	2	6.1
Organizing pneumonia	G3	2	6.1

### 3.2 Comparison of ^68^Ga-FAPI tracer uptake in pulmonary lesions

**Figure 1:**
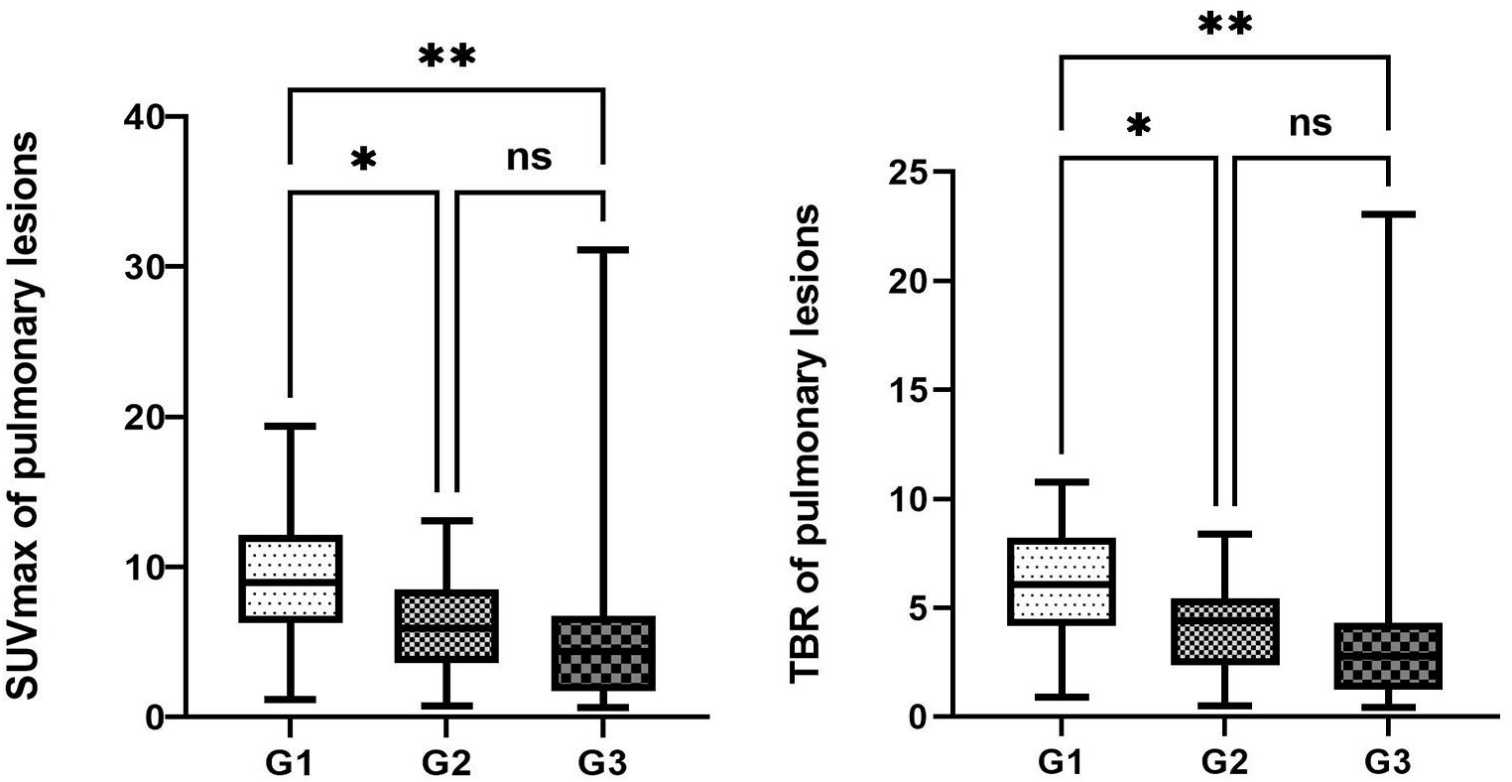
Comparison of the uptake activity of lung lesions and thoracic lymph nodes among the three groups. The legend shows the comparison of uptake activity TBR and SUVmax for lung lesions among the three groups, as well as the comparison of FAPI uptake activity SUVmax and TBR for thoracic lymph nodes among the three groups. *denotes a P-value less than 0.05, **denotes a P-value less than 0.01, and ns represents a non-significant comparison between the two groups.

The maximum SUVmax values for pulmonary lesions across the three groups were observed in non-small cell lung cancer (G1, SUVmax 16.3), malignant pleural mesothelioma with pulmonary metastasis (G2, SUVmax 11.6), and bacterial pneumonia (G3, SUVmax 15.5), indicating differences in ^68^Ga-FAPI uptake among the groups (*P*<0.05, **Table 3**). In pairwise comparisons, the primary lung tumor group (G1) exhibited significantly higher average SUVmax and TBR compared to the metastatic lung tumor group (G2) (Average SUVmax [G1 vs. G2: 9.1 ± 4.1 vs. 6.1 ± 4.1, *P*<0.05]; average TBR [G1 vs. G2: 6.2 ± 2.4 vs. 4.1 ± 2.2, *P*<0.01]). Additionally, G1 showed a higher true positive rate in detecting pulmonary lesions than G2 (95.1% vs. 88%). When comparing G1 with the benign group (G3), G1 demonstrated significantly increased radiotracer activity and true positive rates for detecting pulmonary lesions (average SUVmax [G1 vs. G3: 9.1 ± 4.1 vs. 5.3 ± 5.8, *P*<0.05]; average TBR [G1 vs. G3: 6.2 ± 2.4 vs. 3.2 ± 2.7, *P*<0.01]; true positive rates [G1 vs. G3: 95.1% vs. 75.6%]). However, no significant differences were observed in the uptake capacity of ^68^Ga-FAPI and the true positive rates for pulmonary lesions between G2 and G3 (*P*>0.05, **Figure 1**).

**Table 3:** Comparison of the uptake activity of lung lesions between groups.

Groups	Pulmonary Lesions		
	Mean SUVmax	Mean TBR	True Positive Rate
Primary lung tumor group (G1)	9.1±4.1	6.2±2.4	95.1%(39/41)
Metastatic lung tumor group (G2)	6.1±4.1	4.1±2.2	88.0%(22/25)
The benign group (G3)	5.3±5.8	3.2±2.7	75.6%(25/33)
P^a^	<0.05	<0.01	0.275
P^b^	0.204	0.206	0.202
P^c^	<0.05	<0.01	<0.05
P	<0.05	<0.05	>0.05

^a^P: Comparison between groups G1 and G2; ^b^P: Comparison between groups G1 and G3; ^c^P: Comparison between groups G2 and G3

### 3.3 Comparison of the uptake activity of mass-like lesion and nodular-like pulmonary lesions between groups

The basic morphological characteristics of the size in pulmonary lesions present that the mass lesions are found in 19 cases, accounting for 19.2% (19/99), with the majority originating from primary lung lesions of non-small cell lung cancer (9/41, 21.9%).However, for Mass-like pulmonary lesions (d>3cm), there was no significant difference in the comparative study of the uptake ability of ^68^Ga-FAPI PET/CT among the three groups. And there were 80 cases of nodular-like pulmonary lesions (d<3cm). The distributions of the three groups are as follows: G1 (32/41, 78.1%), G2 (20/25, 80.0%), and G3 (28/33, 84.8%). There are differences in the ^68^Ga-FAPI absorption capacity in the nodular-like pulmonary lesions among three groups (**Table 4**). In pairwise comparisons among the three groups, G1 manifests greater uptake capacities in the nodular-like pulmonary lesions compared to other groups (SUVmax [G1vs G2vs G3: 8.8 ± 4.3vs 5.2 ± 3.2vs 4.9 ± 6.1], p<0.01; TBR [G1vs G2vs G3: 5.7 ± 2.7vs 3.7 ± 2.1vs 3.3 ± 4.4], p<0.05). Nevertheless, there are no remarkable differences in the ^68^Ga-FAPI uptake capacity of nodular-like pulmonary lesions between the G2 and G3 (P>0.05).

**Table 4:** Comparison of the uptake activity of mass-like lesion and nodular-like pulmonary lesions between groups.

Groups	Mass-like lesion(d>3cm)		Nodular pulmonary lesions (d<3cm)	
	The Mean SUVmax	The Mean TBR	The Mean SUVmax	The Mean TBR
Primary lung tumor group (G1)	10.6±2.0	7.5±1.7	8.8±4.3	5.7±2.7
Metastatic lung tumor group (G2)	10.1±1.5	6.0±1.1	5.2±3.2	3.7±2.1
The benign group (G3)	7.8±3.5	6.2±3.1	4.9±6.1	3.3±4.4
P^a^	0.702	0.211	0.012	0.032
P^b^	0.053	0.281	0.003	<0.01
P^c^	0.154	0.869	0.814	0.688
P	0.138	0.359	<0.01	0.012

^a^P: Comparison between groups G1 and G2; ^b^P: Comparison between groups G1 and G3; ^c^P: Comparison between groups G2 and G3.

### 3.4 Comparison of ^68^Ga-FAPI tracer uptake and morphological features

The results of lesion margin characteristics indicated that in Group G1, lesions with smooth margins constituted 24.4% (10/41). In comparison, the corresponding figures for the other two groups were 80.0% (20/25) for G2 and 45.5% (15/33) for G3. Utilizing the smoothness of lesion margins as a morphological criterion, differences in tracer activity among the three groups were further analyzed. The findings revealed no significant differences in tracer uptake for pulmonary lesions with smooth margins across the three groups (*P*>0.05, **Table 5**). Conversely, a significant variance in 68Ga-FAPI activity was observed for pulmonary lesions with non-smooth margins among the groups (*P*<0.01). Pairwise comparisons highlighted that both G1 and G2 exhibited higher Mean SUVmax and Mean TBR for tracer absorption in irregular-bordered pulmonary lesions (SUVmax [G1 vs. G3: 10.9±3.3 vs. 4.6±2.7; G2 vs. G3: 8.5±2.7 vs. 4.6±2.7, *P*<0.01]; TBR [G1 vs. G3: 7.2±2.1 vs. 3.2±2.4; G2 vs. G3: 6.4±1.3 vs. 3.2±2.4, *P*<0.01]). However, between G1 and G2, no significant difference was found in the uptake of the tracer in irregular-bordered pulmonary lesions (*P*>0.05).

**Table 5:** Comparison of the uptake activity of smooth and non-smooth border lung lesions between groups.

Groups	Smooth-bordered pulmonary lesion		Irregular-bordered	
	The Mean SUVmax	The Mean TBR	The Mean SUVmax	The Mean TBR
Primary lung tumor group (G1)	4.5±2.9	3.2±1.9	10.9±3.3	7.2±2.1
Metastatic lung tumor group (G2)	5.7±3.4	3.6±1.9	8.5±2.7	6.4±1.3
The benign group (G3)	3.4±1.8	2.4±1.2	4.6±2.7	3.2±2.4
P^a^	0.287	0.512	0.112	0.463
P^b^	0.125	0.140	<0.01	<0.01
P^c^	0.364	0.257	<0.01	<0.01
P	0.078	0.117	<0.01	<0.01

^a^P: Comparison between groups G1 and G2; ^b^P: Comparison between groups G1 and G3; ^c^P: Comparison between groups G2 and G3.

### 3.5 Correlation analysis of tracer uptake between primary lesions and pulmonary metastases in G2

**Figure 2:**
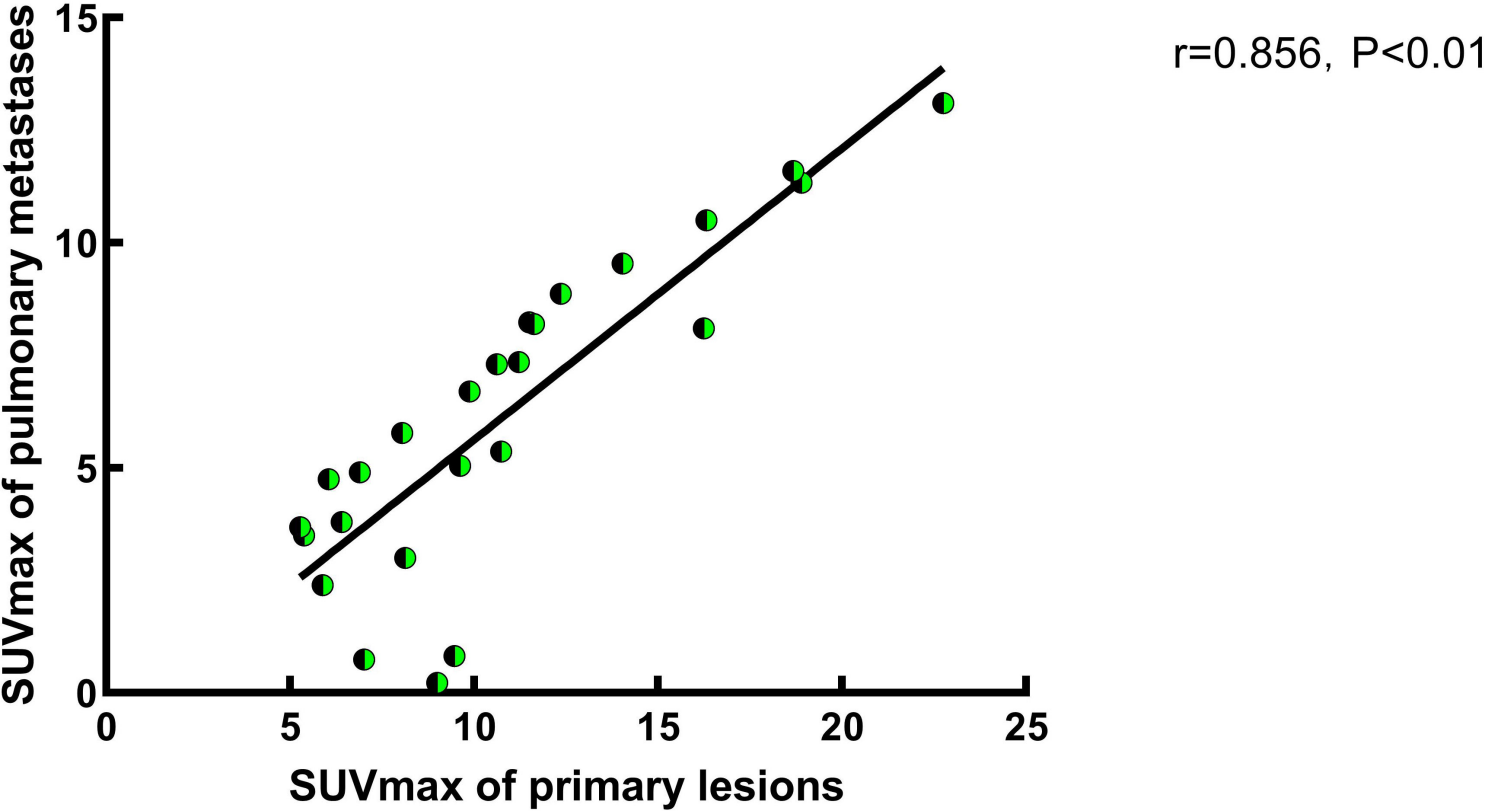
A study on the correlation of SUVmax between primary lesions and pulmonary metastases in Group 2. In Group 2, a correlation analysis of SUVmax between primary and pulmonary metastatic lesions was conducted, and the results indicate a positive correlation between the two. Specifically, the SUVmax of pulmonary metastases increases with the increased FAPI uptake activity of the primary lesion (r=0.856, P<0.05).

A correlation analysis was performed on the SUVmax values of primary tumor lesions and pulmonary metastatic lesions in G2. The analysis revealed a positive correlation between the SUVmax of primary lesions and that of pulmonary metastatic lesions in G2 (**Figure 2**, *P*<0.01). Specifically, an increase in SUVmax of the ^68^Ga-FAPI tracer in primary tumor lesions was associated with a corresponding rise in ^68^Ga-FAPI activity in pulmonary metastatic lesions (r=0.856, *P*<0.05).

## 4 Discussion

In previous clinical practice, ^18^F-FDG PET has shown promise in diagnosing and staging lung cancer (16, 17). However, its non-specificity can lead to false positives, often due to infectious lung diseases’ uptake, with a specificity reaching up to 61% compared to 75% for lung cancer (18). Furthermore, the brain, a common site for lung cancer metastasis, exhibits high ^18^F-FDG uptake, limiting the effectiveness of contrast agents (19, 20). The ^68^Ga-FAPI PET/CT has garnered considerable interest for its superior tumor-to-background ratio across various cancers relative to ^18^F-FDG PET/CT (21). Studies comparing these two imaging modalities have demonstrated that ^68^Ga-FAPI-04 PET/CT identifies more lesions in NSCLC and achieves greater diagnostic accuracy than ^18^F-FDG PET/CT (22). FAP’s extensive expression in lung cancer, notably in SCC (100%) and adenocarcinoma (85.7%), has been confirmed. In cases of early adenocarcinoma, ^18^F-FDG PET/CT only detected three positive lesions, whereas FAP chemical staining positively identified 10 lesions (23), highlighting ^68^Ga-FAPI PET/CT’s potential superiority. Loktev et al. conducted an initial comparison of ^68^Ga-FAPI imaging to ^18^F-FDG in patients with advanced lung adenocarcinoma, revealing significantly higher ^68^Ga-FAPI uptake in metastatic lesions than ^18^F-FDG. Initial assessments often interpreted high ^68^Ga-FAPI uptake in lesions as indicative of malignancy. Yet, with ongoing research, ^68^Ga-FAPI activity has also been detected in a range of conditions, including benign tumors, granulomatous lesions, scars, fibrosis, degenerative changes, inflammatory lesions, and rheumatic diseases (7, 8, 24). Previous studies have shown that ^68^Ga-FAPI PET/CT can distinguish between tumor and inflammatory lesions in organs like the liver (25). For patients with extrapulmonary primary tumors, identifying the nature of new pulmonary lesions is crucial for staging and treatment decisions. Likewise, for those with only pulmonary lesions, determining their nature is essential for guiding clinical management. Therefore, investigating ^68^Ga-FAPI PET/CT’s utility in discerning the nature of pulmonary lesions is of significant clinical importance as a non-invasive diagnostic approach. This retrospective study aimed to analyze ^68^Ga-FAPI tracer absorption in pulmonary lesions of varying natures and morphologies, comparing characteristics across three groups (G1: primary lung tumors, G2: metastatic lung tumors, and G3: benign non-tumor lesions) to assess ^68^Ga-FAPI PET/CT’s value in differentiating the nature of pulmonary occupying lesions.

**Figure 3:**
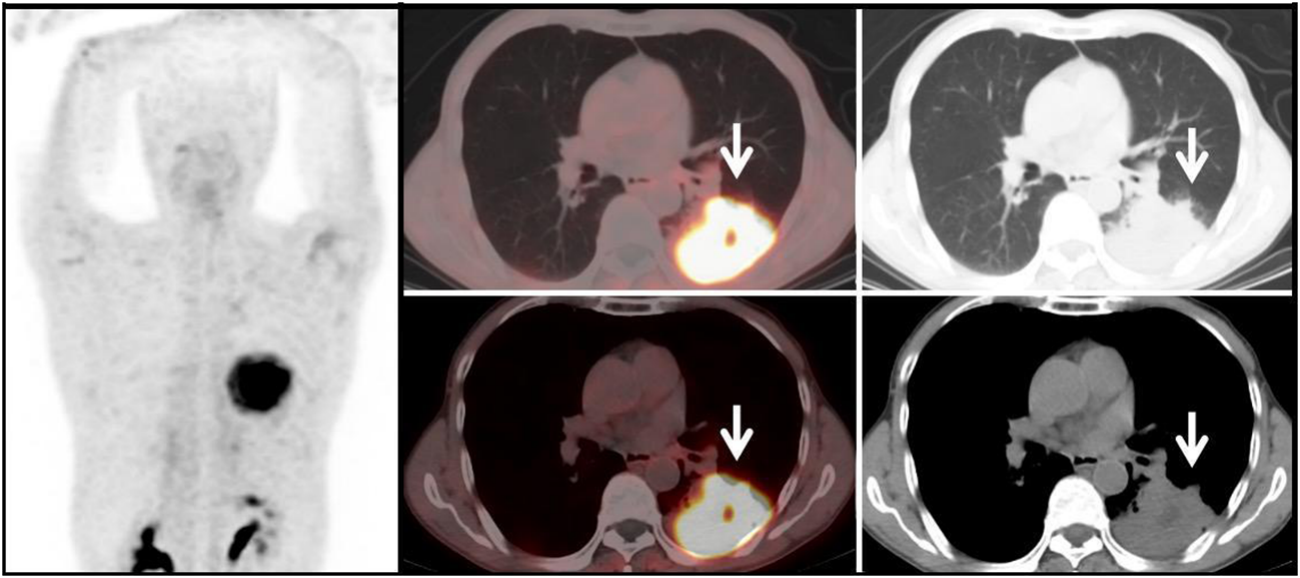
Benign Lesion Group (G3): A 67-year-old male patient with a soft tissue density mass (white arrow) in the left lung, with irregular margins and visible lobulation. Within it, there is a patchy area of decreased density, with significantly increased uptake of ^68^Ga-FAPI tracer, with a SUVmax of approximately 11.6. Histopathological biopsy diagnosis: Organizing pneumonia.

The results of ^68^Ga-FAPI tracer activity across the three groups of pulmonary lesions revealed that Group G1 had the highest mean SUVmax and TBR values (SUVmax: 9.1 ± 4.1, TBR: 6.2 ± 2.4), while Group G3 exhibited the lowest (SUVmax: 5.3 ± 5.8, TBR: 3.2 ± 2.7). Elevated tracer uptake was also observed in lesions from organizing pneumonia (OP) (**Figure 3**) and fibrous proliferative conditions, potentially linked to mesenchymal granulation tissue infiltration and the high expression of fibroblast activation protein within these lesions (26, 27). This increase in ^68^Ga-FAPI activity in pulmonary lesions due to fibroblast proliferation in the pulmonary interstitium was similarly noted in pulmonary fibrosis processes (28). The benign non-tumor pulmonary lesions’ lower mean SUVmax could be attributed to the relatively reduced expression of FAP in pulmonary interstitial fibroblasts compared to tumor-associated fibroblasts. When comparing tracer uptake in pulmonary lesions among the three groups, G1 showed higher imaging agent activity, likely due to the dense presence of tumor-associated fibroblasts and high FAP expression in the Tumor Microenvironment (TME) of primary lung tumors (29). The diminished tracer absorption in G2’s pulmonary lesions might be explained by the primary metastatic lesions originating from thyroid and gastrointestinal (mainly colorectal) cancers, which have moderate ^68^Ga-FAPI uptake capacities (mean SUVmax 6-12) as per Kratochwil et al. (30). Notably, the two primary tumors in G2 with the highest SUVmax were breast cancer and malignant pleural mesothelioma, aligning with the increased tracer uptake reported for these cancers (30). Given the heterogeneity of metastatic and primary tumors, a parallel trend in tracer uptake between pulmonary metastases and primary lesions is plausible. While all three groups demonstrated a relatively high true positive rate for detecting pulmonary lesions, the differences among them were not significant, suggesting the need for further investigation into the utility of the positive detection rate as a diagnostic criterion. In summary, considering the patient’s known malignancy history and the differential ^68^Ga-FAPI tracer uptake in primary tumor lesions, inferences about the origin and nature of pulmonary lesions can be drawn.

**Figure 4:**
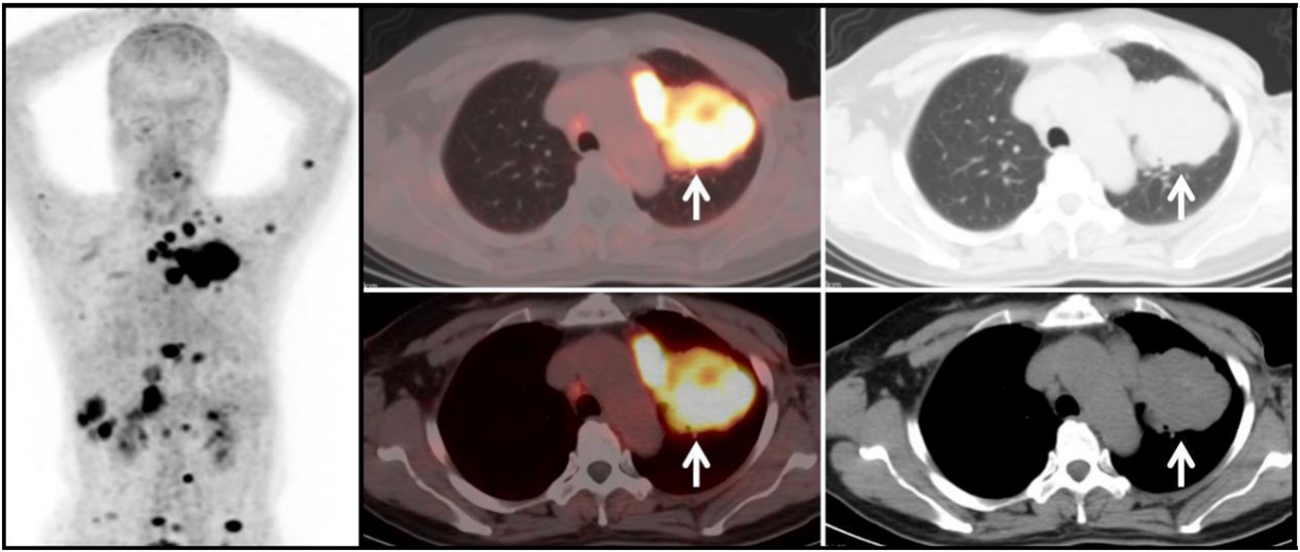
Metastatic Lung Tumor Group (G2): A 51-year-old female patient with a history of breast cancer. A mass-like soft tissue density shadow (white arrow) is observed in the left lung, with relatively regular margins. The lesion shows significantly increased uptake of ^68^Ga-FAPI tracer, with a SUVmax of approximately 11.3. Additionally, there is increased tracer uptake in multiple mediastinal lymph nodes, with a SUVmax of approximately 14.7. Diagnosis based on medical history and subsequent follow-up: Lung metastasis from breast cancer.

**Figure 5:**
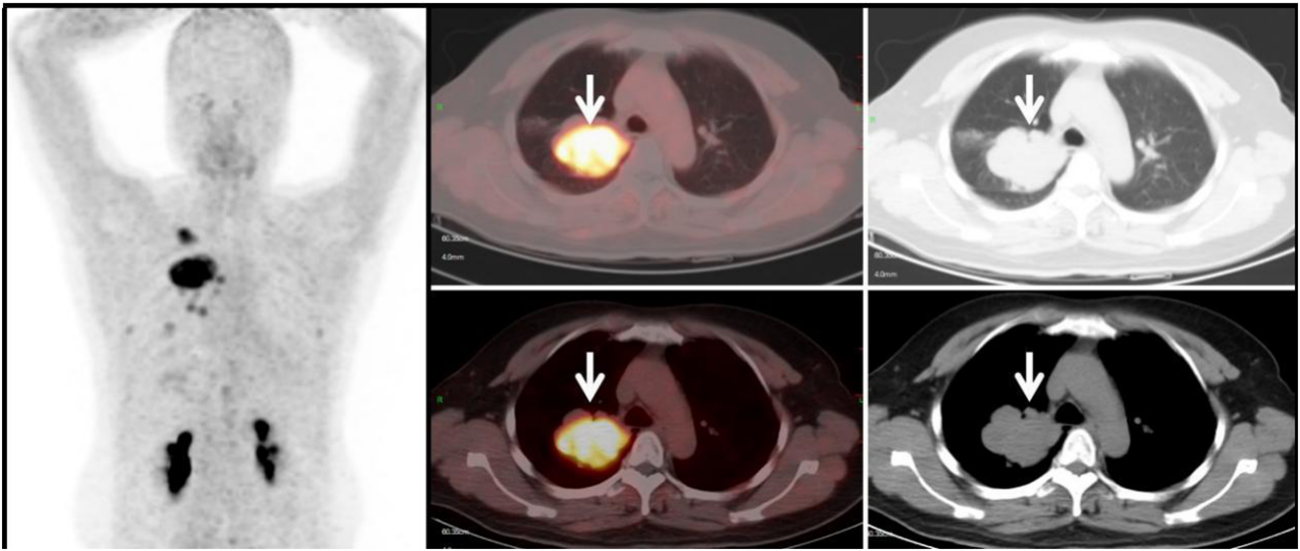
Primary Lung Tumor Group (G1): A 44-year-old female patient with a soft tissue density mass (white arrow) in the right lung. The lesion has irregular margins and visible lobulation, with a patchy area of decreased density within. Additionally, there is significantly increased uptake of 68Ga-FAPI tracer, with a SUVmax of approximately 11.7. Histopathological biopsy diagnosis: Non-small cell lung cancer.

**Figure 6:**
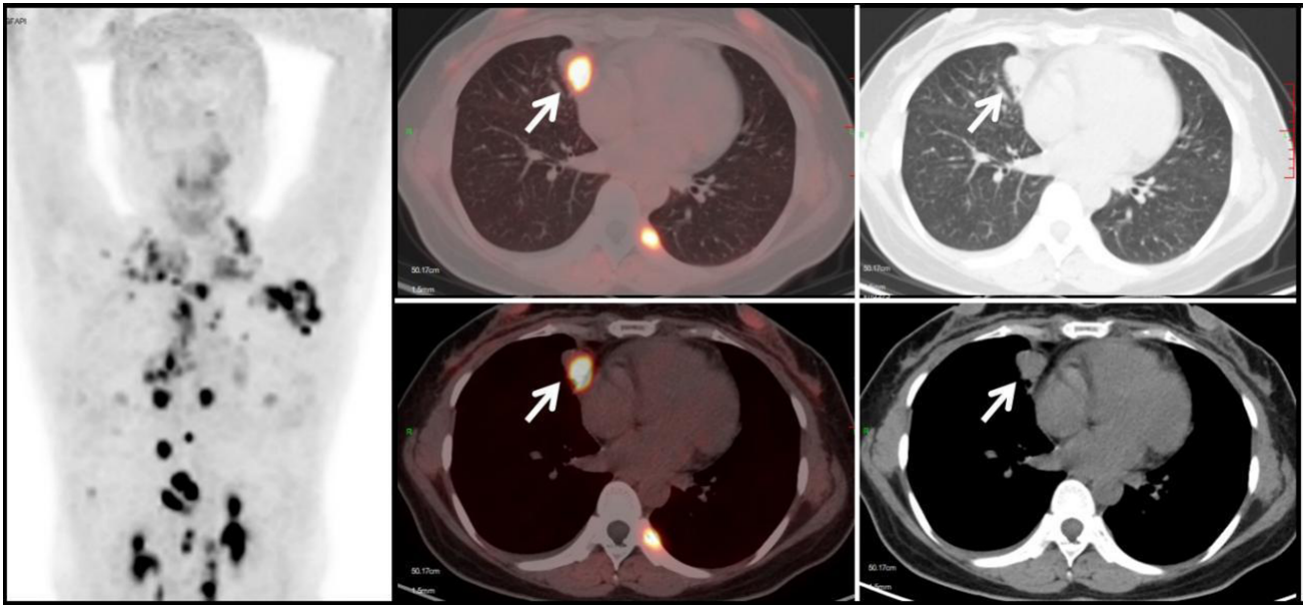
Metastatic Lung Tumor Group (G2): A 53-year-old female patient with a history of cervical cancer. Multiple soft tissue density nodules are observed in the lungs, with the larger ones demonstrated in the axial images (white arrows), with an SUVmax of approximately 10.5. In conjunction with the medical history and subsequent follow-up, the diagnosis is: pulmonary metastasis from cervical cancer.

For the morphological assessment of pulmonary lesions, past scholarship has generally advised against relying solely on individual parameters such as lesion size to determine lesion nature. This caution stems from the observation that some benign, non-neoplastic lung lesions exhibit morphological changes resembling those of malignant tumors, potentially confounding the diagnosis. Consequently, the evaluation of tracer uptake in lesions is essential for clarifying their nature (3, 4, 15, 31). In our research, a comparative analysis was performed on three groups, focusing on variations in lesion size and edge morphology. Lesions were categorized by diameter into nodular lesions (d<3cm) and mass lesions (d>3cm). Among mass-like lesions, no difference in FAPI uptake was observed across the groups. However, for nodular-like pulmonary lesions, Group G1 demonstrated higher FAPI activity compared to the other two groups. These results underscore the utility of ^68^Ga-FAPI PET/CT in differentiating between benign and malignant nodule-like pulmonary lesions, emphasizing its significance in the early detection of primary non-mass-like lung cancer lesions. Prior studies have indicated that pulmonary metastases often feature smooth edges, whereas primary malignant lung tumors typically exhibit irregular edges (32), aligning with the findings regarding smooth-bordered lesions in our study. Moreover, our research revealed no variance in ^68^Ga-FAPI tracer uptake among smooth-bordered lesions across the groups, yet significant differences in FAPI activity for irregular-bordered pulmonary lesions were noted (**Figure 4**). The malignant tumor groups (G1 and G2) exhibited more pronounced imaging agent activity for irregular-bordered lesions than the benign lesion group (G3) (*P*<0.05). For individuals without other primary malignant tumors, FAPI radiotracer activity in irregular-bordered pulmonary lesions holds greater clinical relevance for discerning their benign or malignant nature (**Figure 5**). Consequently, irregular-bordered pulmonary lesions with increased FAPI radiopharmaceutical activity should be more strongly suspected of malignancy, while elevated FAPI uptake in lesions with regular borders does not correlate with their nature. Additionally, our retrospective study discovered a positive correlation between FAPI uptake in metastatic lung tumors and imaging agent activity in their primary lesions (r=0.856, *P*<0.05) (**Figure 6**). Thus, in patients with extrapulmonary primary malignancies manifesting pulmonary lesions, similar FAPI activity levels between the lung lesion and the primary lesion suggest pulmonary metastasis. Conversely, divergent FAPI uptake levels might indicate the presence of a dual-source tumor.

This study is a retrospective analysis and has several limitations, such as the uneven distribution of primary tumor types in the selection of patients with metastatic pulmonary tumors. This may cause bias in lesion uptake. Subsequently, we will further design prospective studies and improve the ratio of tumor types or discuss tumors with different tracer uptake capabilities in groups. Owing to the relatively small sample size and notable selection bias, definitive cutoff values for SUVmaxSUVmax of diverse pulmonary lesions and mediastinal lymph nodes have not been established for the three groups. Future research should aim to resolve this issue. Additionally, the scope of this retrospective analysis was confined to the differentiation of lung lesions via ^68^Ga-FAPI PET/CT.

## 5 Conclusion

In summary, ^68^Ga-FAPI PET/CT imaging has unique value in distinguishing the nature of pulmonary lesions, especially in differentiating primary lung tumors from pulmonary benign lesions. Furthermore, from the combined perspective of morphology and FAPI uptake, ^68^Ga-FAPI PET/CT has particular significance in distinguishing the benign and malignant nature of such irregular-bordered pulmonary lesions. Additionally, the correlation of FAPI uptake levels in pulmonary metastases with those of the primary lesions provides evaluative value. In the future, further comparisons of ^68^Ga-FAPI PET/CT with PET/CT using other tracers can provide a more comprehensive assessment of its value in determining the nature of pulmonary lesions.

## Data availability statement

The original contributions presented in the study are included in the article/supplementary material. Further inquiries can be directed to the corresponding author.

## Ethics statement

The studies involving humans were approved by Ethics Committee of the Affiliated Hospital of Southwestern Medical University (No.AHSWMU-2020-035 and Clinical trial registration No.ChiCTR2100044131). The studies were conducted in accordance with the local legislation and institutional requirements. The participants provided their written informed consent to participate in this study. Written informed consent was obtained from the individual(s) for the publication of any potentially identifiable images or data included in this article.

## Author contributions

YX: Writing – original draft, Writing – review & editing, Data curation, Formal analysis, Investigation, Methodology. WT: Data curation, Investigation, Validation, Writing – review & editing. JM: Conceptualization, Software, Writing – review & editing. YC: Formal analysis, Funding acquisition, Investigation, Project administration, Resources, Supervision, Writing – review & editing.
